# An immunocompetent mouse model of human glioblastoma

**DOI:** 10.18632/oncotarget.17851

**Published:** 2017-05-15

**Authors:** Samantha Semenkow, Shen Li, Ulf D. Kahlert, Eric H. Raabe, Jiadi Xu, Antje Arnold, Miroslaw Janowski, Byoung Chol Oh, Gerald Brandacher, Jeff W.M. Bulte, Charles G. Eberhart, Piotr Walczak

**Affiliations:** ^1^ Department of Pathology, The Johns Hopkins University School of Medicine, Baltimore, MD, USA; ^2^ Russel H. Morgan Department of Radiology and Radiological Science, The Johns Hopkins University School of Medicine, Baltimore, MD, USA; ^3^ Cellular Imaging Section and Vascular Biology Program, Institute for Cell Engineering, The Johns Hopkins University School of Medicine, Baltimore, MD, USA; ^4^ Department of Neurosurgery, Heinrich-Heine-University Dusseldorf, Dusseldorf, Germany; ^5^ Division of Pediatric Oncology, The Johns Hopkins University School of Medicine, Baltimore, MD, USA; ^6^ NeuroRepair Department, Mossakowski Medical Research Centre, PAS, Warsaw, Poland; ^7^ Department of Plastic and Reconstructive Surgery, Vascularized Composite Allotransplantation (VCA) Laboratory, The Johns Hopkins University School of Medicine, Baltimore, MD, USA; ^8^ Department of Ophthalmology, The Johns Hopkins Medical Institute, Baltimore, MD, USA; ^9^ Department of Neurology, Faculty of Medical Sciences, University of Warmia and Mazury, Olsztyn, Poland

**Keywords:** brain tumor, human xenograft, costimulation blockade, immunocompetent, magnetic resonance imaging

## Abstract

Orthotopic xenotransplantation studies represent the final stage in preclinical cancer research and could facilitate the implementation of precision medicine. To date, these xenografts have been tested in immunodeficient animals, but complete elimination of the adaptive immunity is a significant drawback. We present a method of efficient human glioblastoma (GBM) cell engraftment in adult mice with intact immune systems, mediated by a transient blockade of T-cell co-stimulation. Compared to transplants grown in immunodeficient hosts, the resulting tumors more accurately resemble the clinical pathophysiology of patient GBMs, which are characterized by blood-brain-barrier leakage and strong neo-vascularization. We expect our method to have great utility for studying human tumor cell biology, particularly in the field of cancer immunotherapy and in studies on microenvironmental interactions. Given the straightforward approach, the method may also be applicable to other tumor types and additional model organisms.

## INTRODUCTION

**Figure 1:**
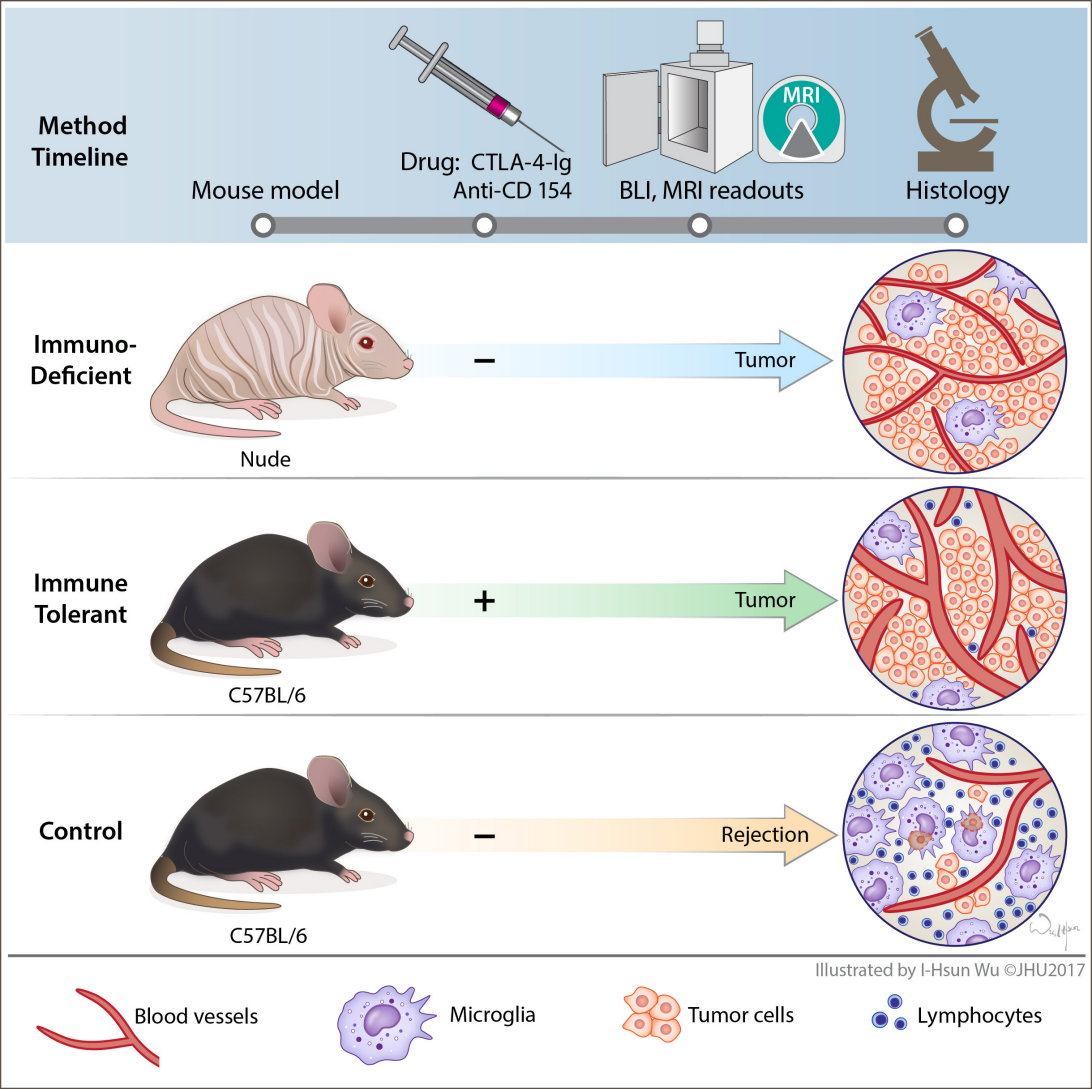
Schematic presentation of the experimental work flow All animals received an intracranial transplant of human GBM neurosphere cells. Immune tolerant mice (immunocompetent C57B/6) were treated with the T-cell co-stimulation blockers Abatacept and MR1, at days 0, 2, 4 and 6 post tumor implantation. Control groups of immunodeficient nude mice (nu/nu) and immunocompetent C57B/6 control mice did not receive treatment with co-stimulation blockers. Tumor engraftment was monitored with bioluminescence and MR imaging. Cancer associated neuro-inflammation in the tumor core and periphery was assessed with histology and immunohistochemistry.

Mouse models are indispensable for the study of disease etiology as well as for the development of new therapies and imaging methods. In oncology, orthotopic xenotransplantation studies are the gold standard for the investigation of human tumor cell biology *in vivo* [1]. Given the graft vs. host immune incompatibility, xenografting relies on the use of immunocompromised hosts, such as athymic nude mice. While such models can be used to generate clinically relevant data, they do not comprise the full spectra of the patient scenario, in part because they cannot take into account the importance of the role the immune system plays in tumor progression [2]. Moreover, the lack of an adaptive immune system in immunodeficient mice prevents the study of treatments modulating the anti-tumor immune response. While studying tumor biology in the context of active immune surveillance can be accomplished with syngeneic models (mouse to mouse grafts), or through the induction of intrinsic tumors in transgenic mouse strains, it excludes the use of human tumor cells. Here we present an immunocompetent orthotopic xenograft model of glioblastoma (GBM), the most lethal and prevalent primary malignant brain tumor in adults, capable of overcoming these limitations [3]. We found that the transient blockade of T-cell activation during the inoculation phase of intracranial grafts led to the successful engraftment of tumors in immunocompetent mice without further manipulation of the immune system. We demonstrate the resulting xenografts better recapitulate the pathological characteristics of human GBM compared to xenografts grown in classical immunodeficient hosts. T-cell activation was blocked pharmacologically based on strategies developed in the fields of organ transplantation and autoimmunity [4]. T-cell function is dependent on the tightly regulated balance of co-stimulatory and co-inhibitory signals. Activation requires both the T-cell receptor to engage with the antigenic peptide/MHC complex and the co-activating receptor CD28 to bind with its tandem ligand CD80/CD86 (B7.1/B7.2). To avoid over-reactive immune responses, activated T-cells upregulate the high affinity-inhibiting co-receptor, cytotoxic T-lymphocyte-associated protein 4 (CTLA-4, CD152). CTLA-4 competes with CD28 to bind CD80/CD86 leading to suppression of T-cell activation and consequently to anergy [5]. Abatacept (CTLA-4-Ig) is an FDA approved, selective T-cell co-stimulation inhibitor that comprises an extracellular domain of CTLA-4 fused with a fragment of a modified Fc portion of human IgG1. Abatacept binds to CD80/CD86 and prevents it from binding to CD28, leading to the inhibition of the second activating signal and causing the T-cell to become anergic. A second arm of T-cell co-stimulation is mediated through the interaction of the T-cell ligand, CD154, with CD40 on the surface of antigen presenting cells. This interaction results in the upregulation of CD80/CD86, which then increases the potential for T-cell activation [6, 7]. The anti-CD154 antibody clone, MR1, recognizes murine CD154 and prevents its interaction with CD40 to further limit T-cell stimulation [8]. Applying a four-time-point treatment scheme of Abatacept and MR1, we present a highly efficient orthotopic xenotransplantation protocol for implanting human cancer cells into immunocompetent mice (Figure 1). This technology could become extremely useful in pre-clinical drug studies, including those in immune therapy research, and would permit the study of the tumor microenvironment in immunocompetent transgenic rodents.

## RESULTS

### Induction of human xenografts in immune tolerant mice

It has been hypothesized that naturally occurring immunological plasticity in the perinatal period can be exploited to induce tolerance to human stem cell xenotransplants in rodents [9]. This phenomenon, however, has not been shown to be reproducible [10, 11]. Despite this, we implanted GBM neurosphere cells into the brains of eleven neonatal mice less than 24 hours old. Five of the eleven mice showed limited tumor growth after several weeks that was detectable by *in vivo* bioluminescence imaging. Two of these mice lost their bioluminescence signal prior to being sacrificed and had no histologically detectable tumors. The remaining three mice had very small, histologically measureable tumors that were extensively infiltrated by neutrophils (data not shown). Neutrophilic infiltrates have been reported in a subset of untreated human GBM using sensitive immunohistochemical detection, but these acute inflammatory cells in our murine tumors appeared much more prominent [12, 13].

**Figure 2:**
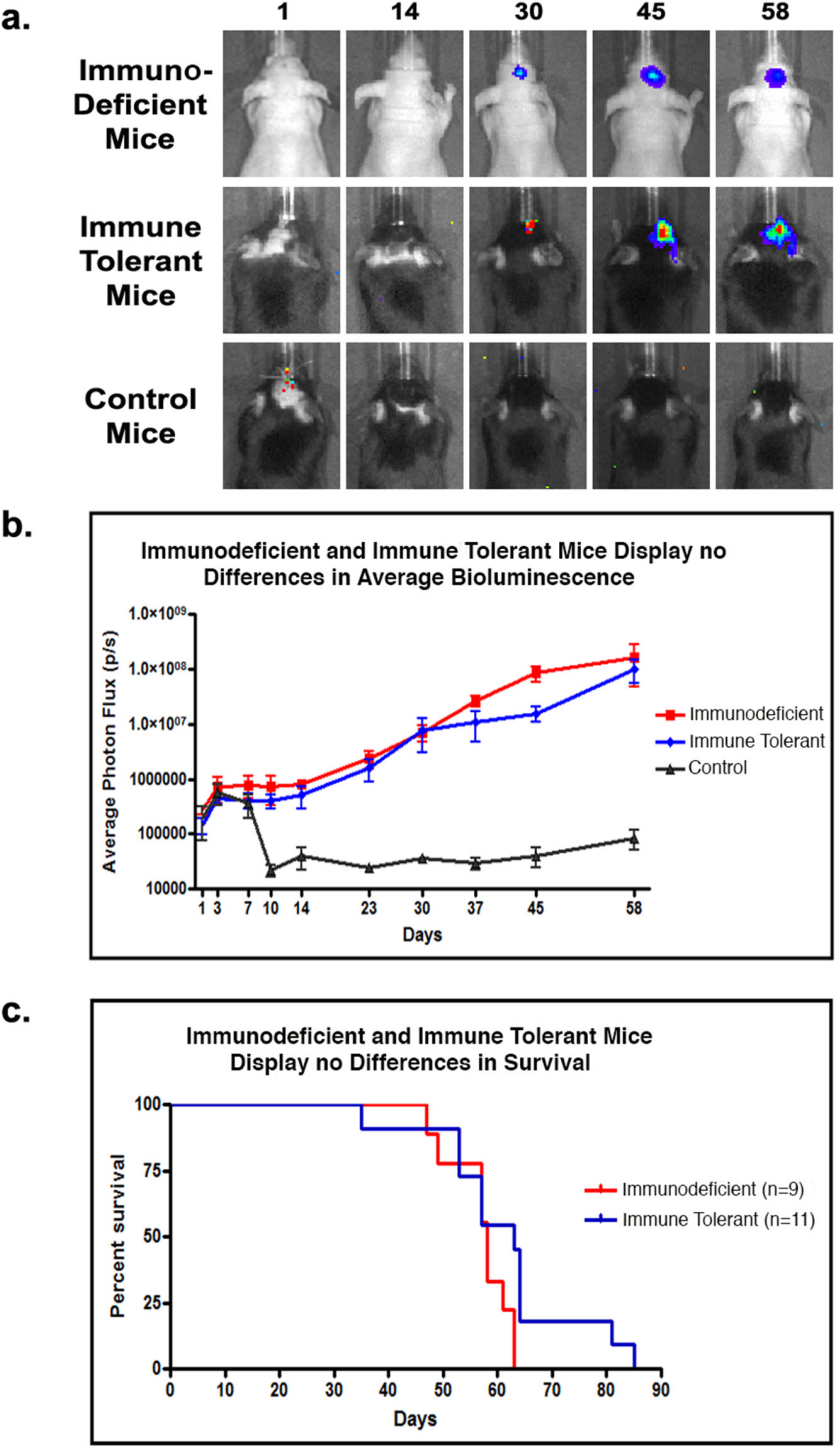
T-cell co-stimulation blockade facilitates human tumor cell engraftment in immune tolerant mice **(a)** Representative bioluminescent imaging for immunodeficient, immune tolerant, and control mice inoculated with human GBM xenografts. **(b)** Mean bioluminescence intensity for each time point per group (n=5 animals/group) showing stable graft establishment only in immune tolerant and immunodeficient mice. **(c)** Kaplan Meyer curve analysis of survival for immunodeficient (n=9) and immune tolerant mice (n=11).

We tested whether immunosuppression with dexamethasone (Dex) would be sufficient to support the growth of human GBM xenografts, reduce inflammation and prevent xenograft rejection. Implantation of GBM neurosphere cells into the brains of neonatal Dex-immunosuppressed mice resulted in the initial survival of small, well-circumscribed transplants. However, all tested animals rejected the graft after five weeks, resulting in the loss of the *in vivo* bioluminescence signal between days 34 and 44 post-inoculation, as shown in a representative litter (Supplementary Figure 1a). While the neutrophilic infiltrate in these was somewhat less prominent than in mice not receiving Dex, it was still significant. Microglia, leukocytes and T-cells were also found to be present in the tumor core (Supplementary Figure 1b). Our experiments suggest that sustained rodent neonatal immune tolerance does not apply to orthotopically implanted GBM cells, and that rejection includes a neutrophilic response not commonly seen in patients. Next, we sought to combat T-cell recruitment at the initial time of implantation of human tumor cells by co-administrating the immune checkpoint blockers Abatacept and MR1. Adult C57B/6 mice implanted intracranially with GBM neurosphere cells received four intraperitoneal injections of the combined co-stimulation blockers at days 0, 2, 4, and 6 post-implantation (hereafter termed immune tolerant mice). For controls, we established transplantation groups with athymic nu/nu mice (hereafter termed immunodeficient mice) and C57B/6 immunocompetent mice (hereafter termed control mice). Both control groups received no drug treatments. Weekly *in vivo* bioluminescence imaging demonstrated similar xenograft growth for immune tolerant and immunodeficient mice (Figure 2a, 2b). Around day 60, both immune tolerant and immunodeficient mice displayed similar tumor burdens and began to exhibit neurological deficits. Conversely, control immunocompetent mice began rejecting the tumor between days 7 and 10, with complete rejection occurring by day 14, as demonstrated the sharp decrease in bioluminescence signaling (Figure 2b). Kaplan Meyer curve analysis shows no significant difference in survival between the immune tolerant and immunodeficient mice (Figure 2c). Immunocompetent control mice did not show any neurological deficits throughout the course of the experiment.

### Immune tolerant tumors superiorly recapitulate the features of human GBM pathophysiology

**Figure 3:**
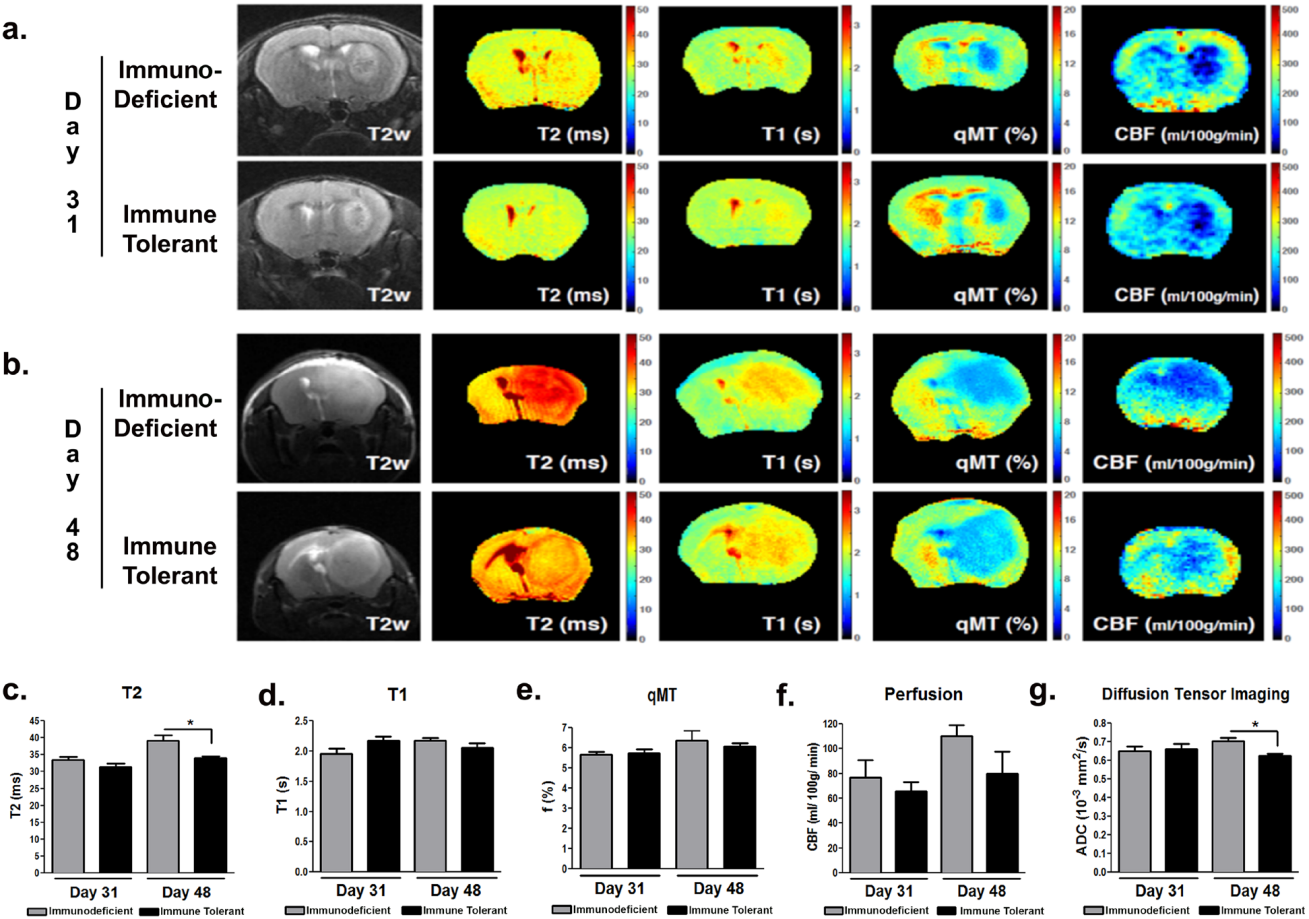
Tumor xenografts in immune tolerant and immunodeficient mice have similar growth characteristics MRI assessments were performed during the xenografts´ most rapid growth phase at day 31 **(a)** and day 48 **(b)** post-inoculation. Similar imaging properties, including quantitative magnetization transfer qMT **(c)**, T2 **(d)**, T1 **(e)**, perfusion **(f)**, and diffusion tensor imaging DTI **(g)**, were detected between both groups. Moderate but statistically significant differences were observed with T2 and DTI at day 48. p<0.05.

Given the exponential growth rate of GBM cells, we monitored the phase of the most rapid progression of xenograft growth with longitudinal magnetic resonance imaging (MRI) at days 31 and 48 post-implantation. Anatomical T2-weighted imaging revealed a similar growth rate in both the immunodeficient and immune tolerant mice (Figure 3a, 3b). Signals in multiparametric, quantitative MRI (quantitative magnetization transfer- qMT, T1, T2, perfusion, and diffusion tensor imaging) were similar between the two groups, showing significant differences at day 48 in T2 (p=0.0301) and diffusion tensor (p=0.016) imaging (Figure 3d, 3g respectively).

**Figure 4:**
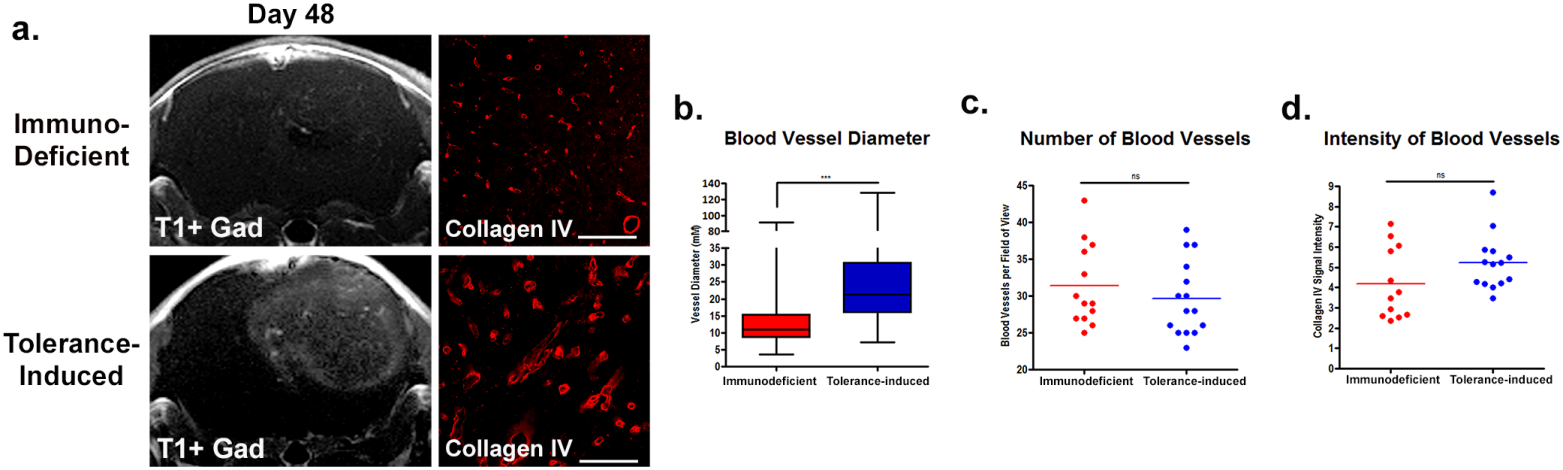
Immune tolerant mice have increased blood-brain-barrier permeability and differences in vascular features Tumor xenografts in immune tolerant mice resulted in stronger and more heterogeneous contrast enhancement on gadolinium-enhanced T1-weighted imaging compared to immunodeficient controls. Stronger contrast enhancement suggested increased blood-brain-barrier permeability/leakage in immune tolerant mice **(a)**. Quantification of histological assessments of the tumor grafts for Collagen IV revealed higher vascularization in transplants grown in mice with intact immune systems, as evidenced by increased blood vessel diameters **(b)**. No differences in blood vessel numbers **(c)** or blood vessel staining intensity **(d)** was observed. Scale bars 50uM, p<0.01.

The tumors in immune tolerant mice showed much lower T2 values, which may be due to higher cellular infiltration and overall lower water content introduced by neuro-inflammatory processes within the tumor at day 48. This was further confirmed by the lower diffusion values in immune tolerant mice compared to the immunodeficient mice, since the self-diffusion coefficient of free water is much higher than that of cellular components. Gadolinium-enhanced T1 imaging showed increased tumor contrast in immune tolerant mice compared to immunodeficient controls, indicating the induction of blood-brain-barrier (BBB) permeability. Heterogeneous imaging signals of the tumor mass also indicated intratumoral variations that recapitulate the biological constitution of human GBM in patients. Moreover, tumors in immune tolerant mice showed increased vascularization in terms of vessel diameter (Figure 4b, p<0.0001), suggesting the importance of an intact immunity for neovascularization. Of note, no difference in the number of vessels (Figure 4c) or vascular Collagen IV staining intensity (Figure 4d) were identified between the two groups.

### Immunogenic xenografts in immune tolerant mice

**Figure 5:**
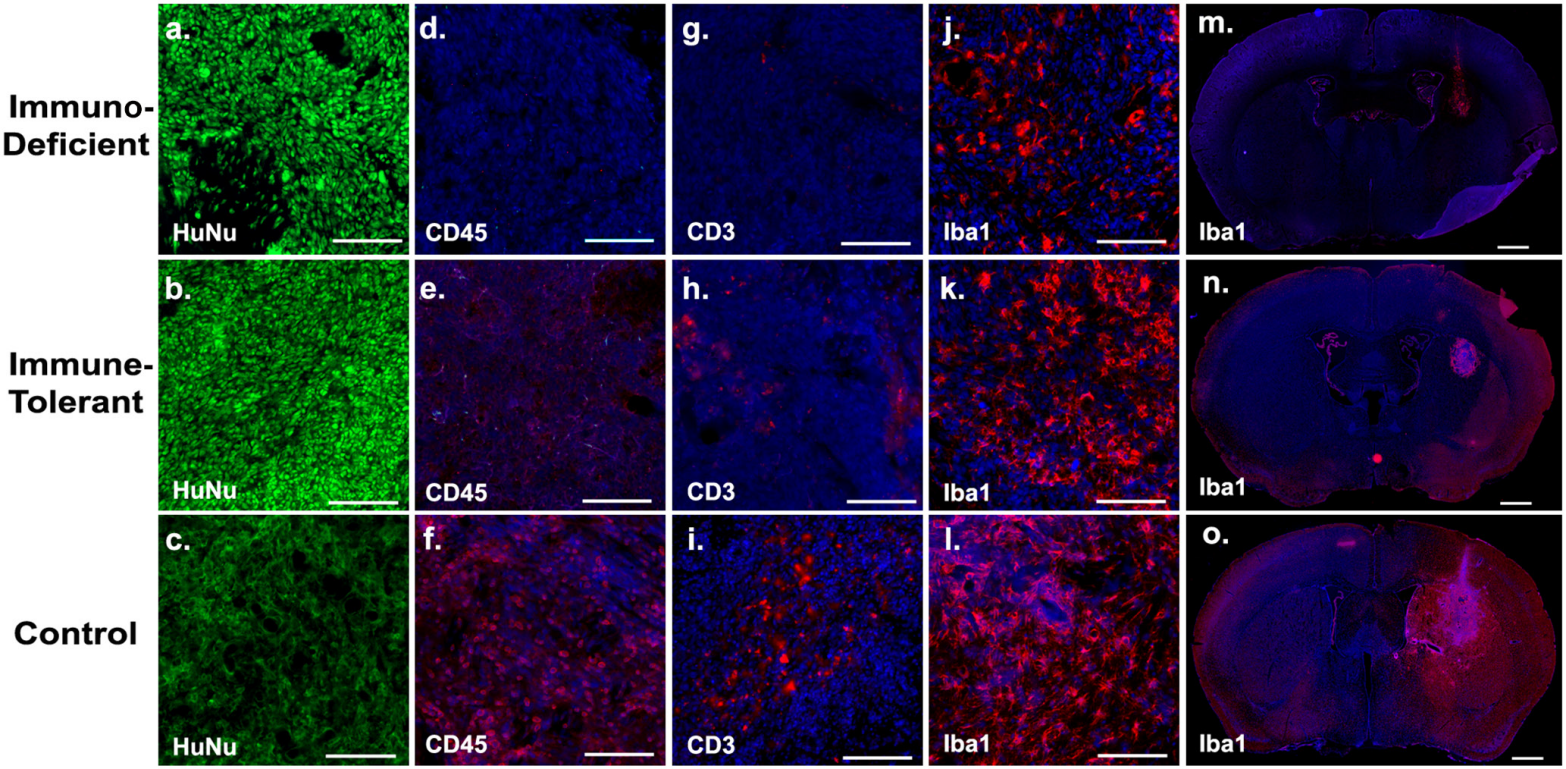
Human xenograft rejection is mediated by T-cells To assess immune cell activation at the grafting site, the brains of the animals from all groups were subjected to immunohistochemistry at day 12 post-transplantation. Growth of human tumor tissue was evidenced by immunoreactivity for Human Nuclear Antigen (HuNu; **a-c**). Xenografts in immune tolerant mice engaged in innate and adaptive immune cell surveillance, as revealed by leukocyte (CD45; common leukocyte antigen; **d-f**), T-cell (CD3; **g-i**), and microglia (Iba1; **j-o**) infiltration. Non-treated, immunocompetent control C57B/6 mice showed massive immune reactivity of all cell types and particularly drastic microglia activation in the entire ipsilateral hemisphere (**o**). Immunodeficient mice showed microglia activation at the tumor core only (**j**). Scale bars are 50uM (subpanels on the left) and 200uM (subpanels on the far right).

To further analyze differences in immune cell recruitment to the tumor site at the time of xenograft rejection, we performed an immunofluorescence evaluation of the brains of the different animal groups (Figure 5). Immune tolerant and immunodeficient mice showed strong staining of Human Nuclear Antigen (HuNu), proving effective tumor engraftment (Figure 5a-5c). The xenografts of all groups showed microglia infiltration (Iba1) in the tumor core (Figure 5d-5f), with marked activation in immunodeficient mice and increased activation in immune tolerant mice. Control immunocompetent mice showed the highest activation of microglia in the tumor core and throughout the entire lateral hemisphere, indicating exorbitant innate immune activation in those animals (Figure 5m-5o). When we compared the tumors of immune tolerant mice to those from immunodeficient animals, we detected increased infiltration of leukocytes (CD45) (Figure 5g-5i) and T-cells (CD3) (Figure 5j-5l), indicating active innate and adaptive immune effectors in the grafts of the C57B/6 mice. In addition, no significant neutrophil infiltration was noted in these lesions. These data strongly indicate that xenograft rejection is mediated by T-cells, which can be overcome by inhibiting T-cell co-stimulation pharmacologically.

## DISCUSSION

Our immunocompetent *in vivo* model for human cancer orthotopic xenografting is a dramatic improvement over current xenograft models. Initially, we demonstrated that orthotopic human brain tumors can effectively be grown in mice with intact immune systems through the pharmacologically mediated transient blockade of T-cell co-activating signals. Although the central nervous system is considered a privileged environment with reduced immunologic surveillance, the potential of the immune system to fight primary brain tumors and cerebral metastases has become increasingly appreciated [14–17]. To date, glioma biology was typically studied in xenografts in immunosuppressed recipients [18], immunodeficient hosts, syngeneic transplantation models, and models of induced intrinsic tumor formation in transgenic mouse strains [19]. Important development towards studying tumors in clinically-relevant setting was establishing human immune system in immunodeficient mice, so called “humanized mice”[20]. While humanized mice are undeniably useful for testing human-specific immunotherapeutics, that system is complex, costly and precludes the use of existing genetically modified immunocompetent mice. There has been one previously reported observation of spontaneous immune tolerance against an adherently grown human GBM cell line in adult SWR/J mice [21]. However, the authors did not provide reasons for the absence of the species-dependent host vs. graft rejection in their model and their experiments were terminated at day 14. The conclusions from their study did not demonstrate whether long-term xenograft growth could be achieved. In contrast, our study demonstrates the successful engraftment and growth of human tumor cells in immunocompetent mice over an extended time period of up to 80 days (Figure 2). We have presented the first mechanistically proven protocol that enables an *in vivo* assessment on the influence of the host’s immune system on xenografted human brain tumor cells.

For some malignant tumors, nearly half the tumor mass can be composed of non-cancerous cells, such as infiltrating leukocytes, which can significantly influence tumorigenesis and the efficacy of chemotherapy [22]. Our histological analysis in immune tolerant mice demonstrated a substantial recruitment of microglia, leukocyte, and T-cells at the tumor site (Figure 5). This suggests an engagement of both the innate and adaptive immune responses during gliomagenesis, an effect that is impossible to obtain in immunodeficient animals. With our presented protocol we are able to generate human tumor xenografts in hosts with intact immune systems. In addition, we have demonstrated that the presence of an intact immune system led to an increase in BBB permeability, as evidenced by contrast-enhanced MRI. We hypothesize that our human GBM *in vivo* mouse model more accurately recapitulates the clinical scenario of a disrupted BBB observed in patients [23]. The activation of the innate immune response, through activated microglia or immunogenic reactivity mediated by cytokines, has been reported to induce BBB dysfunction [24, 25]. In addition to its use for diagnostic and therapy surveillance purposes, increased BBB permeability is extremely important for *in vivo* pre-clinical drug studies since increased permeability could permit greater quantities of chemotherapy drugs access to the tumor core. Therefore, our tumor model may significantly influence the outcomes of preclinical compound tests *in vivo*. We observed that tumors grown in immune tolerant mice had wider blood vessel diameters compared to those developed in immunodeficient mice, and the co-interaction between the immune response and vessel development in tumors has been previously reported [26] (Figure 4). The presence of hypoxic regions, a hallmark of GBM pathology, not only induces angiogenesis, but also creates an immune tolerant environment through secreting factors [27]. Low oxygen tension has been reported to recruit regulatory T-cells that not only induce tolerance of the host immune system to cancer cells, but also stimulate tumor vessel progression [28]. The necessity of an intact immune system for the physiological recapitulation of tumor angiogenesis *in vitro* and *in vivo* has been described for a variety of tumors, including GBM [29, 30]. In addition, the dense vessels in the tumor parenchyma are associated with a greater malignancy of glial tumors, and patients with strongly perfused GBM have a shortened event-free survival [31, 32]. Since GBM is one of the most vascularized brain tumors, the reduction of tumor vascularization has become a central element for the treatment of these malignant tumors, and our model represents a platform for further drug development in this area [33].

The importance of CTLA-4 promoted T-cell immune reactivity for glioma defense has been elaborated both preclinically and clinically [14, 34–37]. In our model, we inhibited T-cell activation through blocking co-stimulation based TCR activation, a strategy that has successfully reduced rejection of allogenic kidney transplants in the clinic [38, 39]. We have now shown, for the first time, that this approach can facilitate graft acceptance between two different mammalian species. Additionally, we expect our protocol could form the basis for subsequent studies transplanting cells of human origin into additional model organisms. Given the half-life of Abatacept of 90 hours and MR1 of 12 days, as measured in mouse serum, we hypothesize that at three weeks after xenotransplantation almost all of the biological functionality of the two reagents could be cleared [40, 41]. A mechanistic explanation on how anergic T-cells become reactivated, after withdrawal of co-stimulation blocking agents, and recruited into the tumor core needs to be elucidated. However, in this study, the immune tolerant and immunodeficient mice succumb to their tumor burden within two months post implantation, and this short timeline was not ideal for conducting these studies. Regardless, it should be noted that despite the presence of both innate and adaptive immune cells within the tumor core of immune tolerant mice, we observed no regression in tumor size by either *in vivo* bioluminescence imaging or MRI. Given the recapitulations of clinical relevance in terms of immunological and perfusion characteristics, we propose that our immune tolerant mouse model has important advantages in cancer research compared to classical xenograft models in immunodeficient hosts. We foresee its utility and application for studies on tumor immunology, intracerebral and intravascular drug delivery, as well as studies on tumor-stroma interaction. For example, our model permits direct injection of human tumor cells into transgenic mice with modified genes in stroma cells thought to regulate tumor growth, circumventing the need for lengthy and expensive animal cross-breeding. Our model would also be appropriate for preclinical studies of cancer vaccines, including those based on RNA particles [35, 36]. Furthermore, we hypothesize that our method is potentially applicable to other model organisms, such as rats or pigs, as well as to *in vivo* tumor models from additional tumor types in mice.

## MATERIALS AND METHODS

### Cell culture and intracranial xenografts

All animal experiments were performed in accordance and were approved by The Johns Hopkins Animal Care and Use Committee.

HSR-GBM-1-Luc neurospheres were dissociated into single cells and suspended in PBS at a final concentration of 1x10^5^/μL [42, 43]. For neonatal inoculations, mouse pups were cryo-anesthetized and 2x10^5^ GBM-1-Luc cells were injected into the right striatum within 24 hours of birth. Pups were weaned at p21. Dexamethasone (Sigma) was injected into pups starting at p7 three times per week (Monday, Wednesday, Friday; 0.3mg/kg I.P.). For T-cell co-stimulatory blockade experiments, C57B/6 adult mice, 4-8 weeks of age were positioned in a stereotaxic frame and 2x10^5^ cells were injected into the right striatum (AP=0.0; ML=2.3mm; DV=2mm) using a 10μl Hamilton syringe with an attached 31-gauge needle. Hamster anti-mouse-CD154mAb (MR1, BioXcell; Lebanon, NH) and Abatacept (Bristol-Myers Squibb, Princeton, NJ) were administered to the experimental group of C57B/6 mice (500μg each I.P.) on days 0, 2, 4, and 6 (n=17). Control groups of C57B/6 mice (n=17) and athymic (nu/nu) mice (n=15) 4-8 weeks of age received no treatment. Mice were monitored daily for neurological symptoms and weighed weekly.

Multimodal Bioluminescence and MR Imaging For bioluminescence imaging (BLI) of tumor growth rate, mice were injected I.P. with 250μL luciferin (15mg/mL), and images were acquired 5-15 minutes after injection. BLI was quantified (photon flux (p/s)) as previously described [44]. For experiments using mouse pups, the earliest time point the mice were imaged was at p21. For experiments using adult mice and the co-stimulation blockers, Abatacept and MR1, imaging began on day 1 post implantation.

MR imaging (MRI) was performed four and eight weeks after tumor implantation. Animals were anesthetized using 2% isoflurane in airflow, followed by 1% to 1.5% isoflurane during the MRI scan. Mice were placed on a water-heated animal bed equipped with temperature and respiratory control. Respiration was monitored and maintained at 40-50/min. All MRI experiments were performed on a horizontal bore 11.7 T Bruker Biospec system (Bruker, Ettlingen, Germany). A 72 mm quadrature volume resonator was used as a transmitter, and a 2×2 mouse phased array coil was used to acquire MR images. The two coils were provided by Bruker Corporation (Ettlingen, Germany). T2 weighted (T2w) images were acquired using a Rapid Imaging with Refocused Echoes (RARE) sequence, with 30 ms echo time (TE) and 5 s repetition time (TR), a RARE factor of 8, slice thickness (SI) = 1 mm, and a matrix size of 128 × 128. The T_1_ relaxation times of the mouse brain were measured using variable TR (TR= 0.25, 0.69, 1.26, 2.06, 3.41, 10s) sequences with RARE readout with TE 20 ms, matrix 96 × 96, SI 1mm, and a RARE factor of 8. The T2 map was obtained by a multi-slice multi echo (MSME) MRI with geometry identical to that of the T1 map measurement and an echo space of 10 ms. A quantitative magnetization transfer (qMT) method, based on the on-resonance variable delay multi pulse (onVDMP) sequence, was used to extract the macromolecular proton fraction map (f) [45]. The perfusion maps of mice brains were recorded by the Steady Pulsed Imaging and Labeling (SPIL) scheme [46]. Gadolinium (100μL) was injected I.P. for contrast-enhanced T1 scans and T1+gadolinium images were acquired at 5, 10, and 15 minutes post injection. The T1-weighted images were recorded using a spin echo sequence, with a 100 ms repetition time and a 9.1 echo time. The number of averages was 8, with matrix size 128X128. Images were all processed using custom routines written in Matlab (MathWorks, Massachusetts).

### Immunohistochemistry and immunofluorescence

Mice were sacrificed when neurological symptoms became apparent in survival studies and on day 12 for assessing immune cells mediating the rejection of human cells. Following transcardial perfusion with 4% paraformaldehyde, brain tissue was postfixed by either 4% paraformaldehyde for frozen sections or 10% formalin for paraffin-embedded sections.

Immunohistochemistry was performed on de-paraffinized sections. Antigen retrieval was achieved using Antigen Unmasking Solution (Vector Laboratories, Burlingame, CA) and sections were incubated overnight with primary antibodies at 4°C. Sections were incubated with secondary antibody for one hour and immunoreactivity was visualized using DAB. Slides were counterstained in hematoxylin. Primary antibodies used: human specific NESTIN (1:500; Millipore, #MAB5326); IBA1 (1:100; Wako, #019-19741); CD45 (1:150; Bio-Rad, #MCA1388); and CD3 (1:100; Bio-Rad, #MCA1477).

Immunofluorescence was performed on frozen sections, 30μM thick. Sections were blocked using 0.1% Triton and 5% BSA, and incubated overnight with primary antibodies at 4°C. Sections were incubated for one hour with either Alexa-488 or Alexa-594 (Invitrogen, 1:500) secondary antibodies, and were counterstained with DAPI for 10 minutes. Primary antibodies used: IBA1 (1:100; Wako, #019-19741); CD45 (1:100; Bio-Rad, #MCA1388); COLLAGEN IV (1:250; Abcam, ab6586); CD3 (1:200; Bio-Rad, #MCA1477); human-specific NUCLEI (1:250; Millipore, #MAB1281); and STEM123 (1:500; StemCells, #AB-123-U-050).

## SUPPLEMENTARY MATERIALS FIGURE



## Abbreviations

GBM: glioblastoma multiforme
CTLA-4: cytotoxic T-lymphocyte associated protein
Dex: dexamethasone
BBB: blood-brain-barrier
MRI: magnetic resonance imaging
BLI: bioluminescence imaging
qMT: quantitative magnetization transfer
T2w: T2 weighted
RARE: rapid imaging with refocused echoes
TE: echo time
TR: repetition time
SI: slice thickness
MSME: multi-slice multi echo
onVDMP: on-resonance variable delay multi pulse
SPIL: steady pulsed imaging and labeling

## References

[1] Hidalgo M, Amant F, Biankin AV, Budinska E, Byrne AT, Caldas C, Clarke RB, de Jong S, Jonkers J, Maelandsmo GM, Roman-Roman S, Seoane J, Trusolino L, Villanueva A (2014). Patient-derived xenograft models: an emerging platform for translational cancer research. Cancer Discov.

[2] Finn OJ (2012). Immuno-oncology: understanding the function and dysfunction of the immune system in cancer. Ann Oncol.

[3] Lathia JD, Mack SC, Mulkearns-Hubert EE, Valentim CL, Rich JN (2015). Cancer stem cells in glioblastoma. Genes Dev.

[4] Adams AB, Ford ML, Larsen CP (2016). Costimulation blockade in autoimmunity and transplantation: the CD28 pathway. J Immunol.

[5] Krummel MF, Allison JP (1995). CD28 and CTLA-4 have opposing effects on the response of T cells to stimulation. J Exp Med.

[6] Jones KW, Hackett CJ (1996). Activated T hybridomas induce upregulation of B7-1 on bystander B lymphoma cells by a contact-dependent interaction utilizing CD40 ligand. Cell Immunol.

[7] Grewal IS, Flavell RA (1998). CD40 and CD154 in cell-mediated immunity. Ann Rev Immunol.

[8] Larsen CP, Elwood ET, Alexander DZ, Ritchie SC, Hendrix R, Tucker-Burden C, Cho HR, Aruffo A, Hollenbaugh D, Linsley PS, Winn KJ, Pearson TC (1996). Long-term acceptance of skin and cardiac allografts after blocking CD40 and CD28 pathways. Nature.

[9] Kelly CM, Precious SV, Scherf C, Penketh R, Amso NN, Battersby A, Allen ND, Dunnett SB, Rosser AE (2009). Neonatal desensitization allows long-term survival of neural xenotransplants without immunosuppression. Nat Methods.

[10] Janowski M, Jablonska A, Kozlowska H, Orukari I, Bernard S, Bulte JW, Lukomska B, Walczak P (2012). Neonatal desensitization does not universally prevent xenograft rejection. Nat Methods.

[11] Mattis VB, Wakeman DR, Tom C, Dodiya HB, Yeung SY, Tran AH, Bernau K, Ornelas L, Sahabian A, Reidling J, Sareen D, Thompson LM, Kordower JH, Svendsen CN (2014). Neonatal immune-tolerance in mice does not prevent xenograft rejection. Exp Neurol.

[12] Han S, Liu Y, Li Q, Li Z, Hou H, Wu A (2015). Pre-treatment neutrophil-to-lymphocyte ratio is associated with neutrophil and T-cell infiltration and predicts clinical outcome in patients with glioblastoma. BMC Cancer.

[13] Fossati G, Ricevuti G, Edwards SW, Walker C, Dalton A, Rossi ML (1999). Neutrophil infiltration into human gliomas. Acta Neuropathol.

[14] Muldoon LL, Alvarez JI, Begley DJ, Boado RJ, Del Zoppo GJ, Doolittle ND, Engelhardt B, Hallenbeck JM, Lonser RR, Ohlfest JR, Prat A, Scarpa M, Smeyne RJ (2013). Immunologic privilege in the central nervous system and the blood-brain barrier. J Cereb Blood Flow Metab.

[15] Wainwright DA, Chang AL, Dey M, Balyasnikova IV, Kim CK, Tobias A, Cheng Y, Kim JW, Qiao J, Zhang L, Han Y, Lesniak MS (2014). Durable therapeutic efficacy utilizing combinatorial blockade against IDO, CTLA-4, and PD-L1 in mice with brain tumors. Clin Cancer Res.

[16] Berghoff AS, Kiesel B, Widhalm G, Rajky O, Ricken G, Wohrer A, Dieckmann K, Filipits M, Brandstetter A, Weller M, Kurscheid S, Hegi ME, Zielinski CC (2015). Programmed death ligand 1 expression and tumor-infiltrating lymphocytes in glioblastoma. Neuro-oncology.

[17] Berghoff AS, Preusser M (2015). The inflammatory microenvironment in brain metastases: potential treatment target?. Chin Clin Oncol.

[18] Qin H, Janowski M, Pearl MS, Malysz-Cymborska I, Li S, Eberhart CG, Walczak P (2017). Rabbit model of human gliomas: implications for intra-arterial drug delivery. PLoS One.

[19] Oh T, Fakurnejad S, Sayegh ET, Clark AJ, Ivan ME, Sun MZ, Safaee M, Bloch O, James CD, Parsa AT (2014). Immunocompetent murine models for the study of glioblastoma immunotherapy. J Transl Med.

[20] Ashizawa T, Iizuka A, Nonomura C, Kondou R, Maeda C, Miyata H, Sugino T, Mitsuya K, Hayashi N, Nakasu Y, Maruyama K, Yamaguchi K, Katano I (2017). Antitumor effect of programmed death-1 (PD-1) blockade in humanized the NOG-MHC double knockout mouse. Clin Cancer Res.

[21] Garcia C, Dubois LG, Xavier AL, Geraldo LH, da Fonseca AC, Correia AH, Meirelles F, Ventura G, Romao L, Canedo NH, de Souza JM, de Menezes JR, Moura-Neto V (2014). The orthotopic xenotransplant of human glioblastoma successfully recapitulates glioblastoma-microenvironment interactions in a non-immunosuppressed mouse model. BMC Cancer.

[22] Quail DF, Joyce JA (2013). Microenvironmental regulation of tumor progression and metastasis. Nat Med.

[23] Dubois LG, Campanati L, Righy C, D'Andrea-Meira I, Spohr TC, Porto-Carreiro I, Pereira CM, Balca-Silva J, Kahn SA, DosSantos MF, Oliveira Mde A, Ximenes-da-Silva A, Lopes MC (2014). Gliomas and the vascular fragility of the blood brain barrier. Frontiers in cellular neuroscience.

[24] da Fonseca AC, Matias D, Garcia C, Amaral R, Geraldo LH, Freitas C, Lima FR (2014). The impact of microglial activation on blood-brain barrier in brain diseases. Front Cell Neurosci.

[25] Banks WA, Erickson MA (2010). The blood-brain barrier and immune function and dysfunction. Neurobiol Dis.

[26] Hendry SA, Farnsworth RH, Solomon B, Achen MG, Stacker SA, Fox SB (2016). The role of the tumor vasculature in the host immune response: implications for therapeutic strategies targeting the tumor microenvironment. Front Immunol.

[27] Kaur B, Khwaja FW, Severson EA, Matheny SL, Brat DJ, Van Meir EG (2005). Hypoxia and the hypoxia-inducible-factor pathway in glioma growth and angiogenesis. Neuro Oncol.

[28] Facciabene A, Peng X, Hagemann IS, Balint K, Barchetti A, Wang LP, Gimotty PA, Gilks CB, Lal P, Zhang L, Coukos G (2011). Tumour hypoxia promotes tolerance and angiogenesis via CCL28 and T(reg) cells. Nature.

[29] Fainaru O, Almog N, Yung CW, Nakai K, Montoya-Zavala M, Abdollahi A, D'Amato R, Ingber DE (2010). Tumor growth and angiogenesis are dependent on the presence of immature dendritic cells. FASEB J.

[30] Stockmann C, Schadendorf D, Klose R, Helfrich I (2014). The impact of the immune system on tumor: angiogenesis and vascular remodeling. Front Oncol.

[31] Jain RK, di Tomaso E, Duda DG, Loeffler JS, Sorensen AG, Batchelor TT (2007). Angiogenesis in brain tumours. Nat Rev Neurosci.

[32] Furtner J, Bender B, Braun C, Schittenhelm J, Skardelly M, Ernemann U, Bisdas S (2014). Prognostic value of blood flow measurements using arterial spin labeling in gliomas. PLoS One.

[33] Carmeliet P, Jain RK (2011). Principles and mechanisms of vessel normalization for cancer and other angiogenic diseases. Nat Rev Drug Discov.

[34] Vom Berg J, Vrohlings M, Haller S, Haimovici A, Kulig P, Sledzinska A, Weller M, Becher B (2013). Intratumoral IL-12 combined with CTLA-4 blockade elicits T cell-mediated glioma rejection. J Exp Med.

[35] Kranz LM, Diken M, Haas H, Kreiter S, Loquai C, Reuter KC, Meng M, Fritz D, Vascotto F, Hefesha H, Grunwitz C, Vormehr M, Husemann Y (2016). Systemic RNA delivery to dendritic cells exploits antiviral defence for cancer immunotherapy. Nature.

[36] Melero I, Gaudernack G, Gerritsen W, Huber C, Parmiani G, Scholl S, Thatcher N, Wagstaff J, Zielinski C, Faulkner I, Mellstedt H (2014). Therapeutic vaccines for cancer: an overview of clinical trials. Nat Rev Clin Oncol.

[37] Fecci PE, Ochiai H, Mitchell DA, Grossi PM, Sweeney AE, Archer GE, Cummings T, Allison JP, Bigner DD, Sampson JH (2007). Systemic CTLA-4 blockade ameliorates glioma-induced changes to the CD4+ T cell compartment without affecting regulatory T-cell function. Clin Cancer Res.

[38] Reardon DA, Gokhale PC, Klein SR, Ligon KL, Rodig SJ, Ramkissoon SH, Jones KL, Conway AS, Liao X, Zhou J, Wen PY, Van Den Abbeele AD, Hodi FS (2016). Glioblastoma eradication following immune checkpoint blockade in an orthotopic, immunocompetent model. Cancer Immunol Res.

[39] Vincenti F, Rostaing L, Grinyo J, Rice K, Steinberg S, Gaite L, Moal MC, Mondragon-Ramirez GA, Kothari J, Polinsky MS, Meier-Kriesche HU, Munier S, Larsen CP (2016). Belatacept and long-term outcomes in kidney transplantation. New Engl J Med.

[40] Srinivas NR, Shyu WC, Weiner RS, Tay LK, Greene DS, Barbhaiya RH (1995). Pharmacokinetics of CTLA4Ig (BMS-188667), a novel immunosuppressive agent, following intravenous and subcutaneous administration to mice. J Parma Sci.

[41] Foy TM, Shepherd DM, Durie FH, Aruffo A, Ledbetter JA, Noelle RJ (1993). *In vivo* CD40-gp39 interactions are essential for thymus-dependent humoral immunity. II. Prolonged suppression of the humoral immune response by an antibody to the ligand for CD40, gp39. J Exp Med.

[42] Chu Q, Orr BA, Semenkow S, Bar EE, Eberhart CG (2013). Prolonged inhibition of glioblastoma xenograft initiation and clonogenic growth following *in vivo* Notch blockade. Clin Cancer Res.

[43] Clark AJ, Safaee M, Oh T, Ivan ME, Parimi V, Hashizume R, Ozawa T, James CD, Bloch O, Parsa AT (2014). Stable luciferase expression does not alter immunologic or *in vivo* growth properties of GL261 murine glioma cells. J Transl Med.

[44] Janowski M, Engels C, Gorelik M, Lyczek A, Bernard S, Bulte JW, Walczak P (2014). Survival of neural progenitors allografted into the CNS of immunocompetent recipients is highly dependent on transplantation site. Cell Transplant.

[45] Xu J, Chan KW, Xu X, Yadav N, Liu G, van Zijl PC (2017). On-resonance variable delay multipulse scheme for imaging of fast-exchanging protons and semisolid macromolecules. Magn Reson Med.

[46] Xu J, Qin Q, Wu D, Hua J, Song X, McMahon MT, Northington FJ, Zhang J, van Zijl PC, Pekar JJ (2016). Steady pulsed imaging and labeling scheme for noninvasive perfusion imaging. Magn Reson Med.

