# Supplementary material for: An immunocompetent mouse model of human glioblastoma
Source: Oncotarget. 2017 May 15;8(37):61072–82. doi: 10.18632/oncotarget.17851 (PMC5617407; doi:10.18632/oncotarget.17851)
Supplement: Supplementary file 1 [file oncotarget-08-61072-s001.pdf]

An immunocompetent mouse model of human glioblastoma

SUPPLEMENTARY MATERIALS

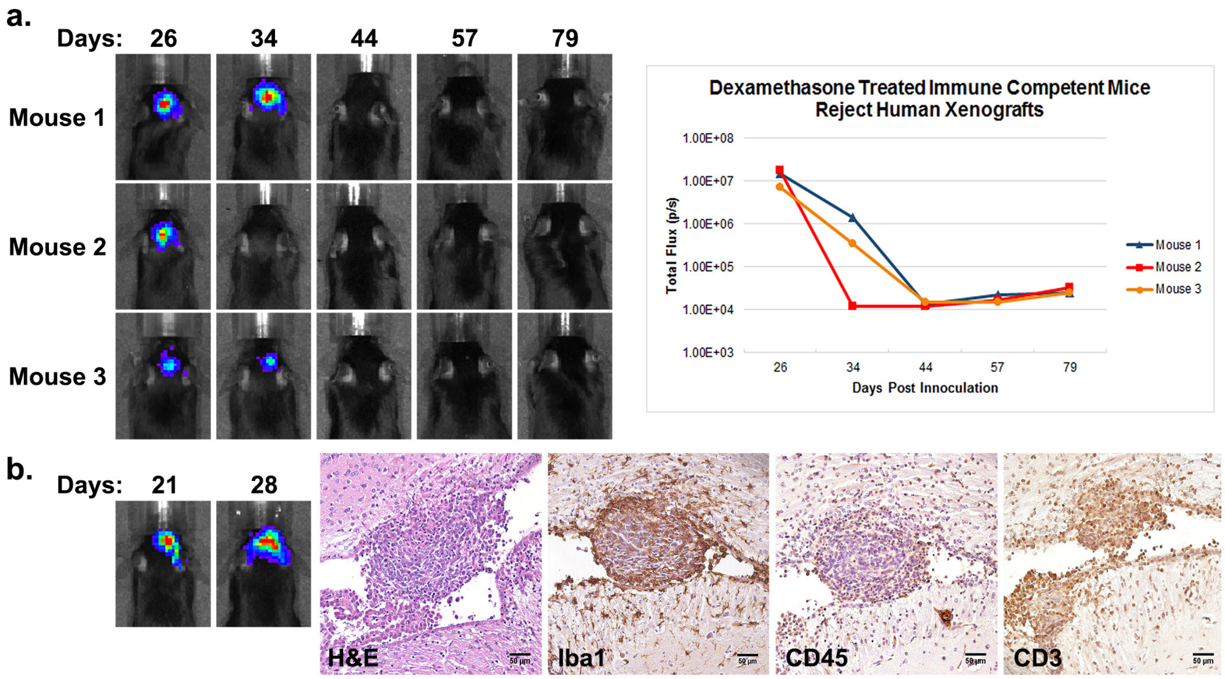

Supplementary Figure 1: Dexamethasone treatment is not sufficient to prevent human xenograft rejection in neonatal mouse pups. (a) Representative bioluminescent imaging of mice inoculated with human GBM neurosphere cells as neonates and treated 3x per week with 0.03mg/kg dexamethasone. Bioluminescence imaging for each mouse over the course of 79 days shows successful initial graft establishment, followed by subsequent xenograft rejection. **(b)** Representative mouse sacrificed after 28 days. Immunohistochemistry on paraffin embedded sections shows the presence of a small tumor infiltrated with microglia (iba1), leukocytes (CD45) and T-cells (CD3). Human Nestin staining confirms the masses are of human origin.
